# Dr Dennis Sifris: In memorium

**DOI:** 10.4102/sajhivmed.v21i1.1143

**Published:** 2020-08-26

**Authors:** David C. Spencer

**Affiliations:** 1 Division of Infectious Diseases, Department of Medicine, Helen Joseph Hospital, University of the Witwatersrand, Johannesburg, South Africa

Dr Dennis Sifris, a pioneer in the field of HIV in South Africa, died in July 2020 of colonic cancer. He was 75 years old.

In 1984, Dennis and the late Professor Reuben Sher established South Africa’s first HIV Clinic at the Johannesburg Hospital (now the Charlotte Maxeke Johannesburg Academic Hospital). The HIV pandemic had come to the attention of the medical community three years earlier because of the deaths of a large number of young men in the United States of America (USA). Homosexuality was banned and prosecuted in South Africa in that era. Dennis and Reuben made it their business to identify the infected and to provide them with care and support. It was at a time when the clinicians faced ridicule or censure from colleagues for their support of homosexuals and the HIV infected.

The lives of multiplied thousands were saved. Health workers have been trained. Medical students have become professors. The practice of HIV medicine has been influenced by South African scientists and researchers who were trained in the clinic. This too, is Dennis’ legacy.

Dennis was loud. He was visible. He had marched with Larry Kramer in New York and Los Angeles and witnessed the birth of the Gay Men’s Health Crisis movement in that country. [Larry died in New York in May this year]. Dennis’ activism was a catalyst in this process. Others such as The AIDS Law Project, the Treatment Action Campaign, the Stop Stock-Outs project, and the Southern African HIV Clinicians’ Society, followed this process. Perhaps our combined action in 2002 and 2003 ensured antiretroviral treatment for all South African citizens and helped to end a denialist presidency.

For much of Dennis’ career, he ran a large private practice in Johannesburg. In 1999, he joined a South African Healthcare Management company (LifeSense HIV Disease Management) as Chief Medical Officer. Private practice ‘had taken its toll’, he said. In this new position he oversaw the care of more than 120,000 people living with HIV. More recently, he moved with his partner (Jamie) to the USA. Dennis is a cofounder of the SA HIV Clinicians’ Society and was active in local HIV-conferences and despite his recent ill health, maintained an interest in all things related to HIV in his patients in South Africa.

Dennis, how should we measure your time with us? Thank you for friendship, for colouring our lives with laughter and for me – surprise. For being impossible (embarrassing) when that was needed. Thank you for caring for the sick. Thank you for the lives you saved. Thank you for accepting graciously my criticism of your clinic notes – not enough detail! Ooh! – when I was chief. Dennis, thank you for giving a human face to HIV and AIDS, and for being yourself. From all of us in the Clinicians’ Society, “Ti sei perso”. (You are missed). And to Jamie, our heartfelt condolences.

DC Spencer, Editor-in-Chief

